# Metabolism of Innate Immune Cells in Cancer

**DOI:** 10.3390/cancers13040904

**Published:** 2021-02-21

**Authors:** Ronan Talty, Kelly Olino

**Affiliations:** 1 Department of Pathology, Yale University, New Haven, CT 06520, USA; ronan.talty@yale.edu; 2 Department of Surgery, Yale University, New Haven, CT 06520, USA

**Keywords:** cancer, metabolism, innate immunity, immunotherapy

## Abstract

**Simple Summary:**

Both cancer cells and immune cells depend on specific metabolic programs for their survival and function. Depending on which metabolic changes occur, immune cells can either promote or suppress the antitumor immune response. This review summarizes the metabolic pathways that polarize innate immune cells for immune activation or suppression and describes the current clinical applications of these findings.

**Abstract:**

Cancer cells possess specific metabolic requirements for their survival, proliferation, and progression. Within a shared microenvironment, immune cells depend on competing metabolic pathways for their development and effector function. As a result, local acidification, hypoxia, and nutrient depletion in the tumor microenvironment can alter the antitumor immune response and even promote resistance to immunotherapies such as immune checkpoint blockade and adoptive cell transfer. Although T cells are the primary effectors of the antitumor response, growing evidence demonstrates that innate immune cells are critical to successful tumor clearance. This review aims to summarize current research related to the innate immune system, metabolism, and cancer. We first discuss the specific metabolic requirements of innate immune cells for immune activation and suppression and conclude by highlighting ongoing clinical applications of these findings.

## 1. Introduction

**Figure 1:**
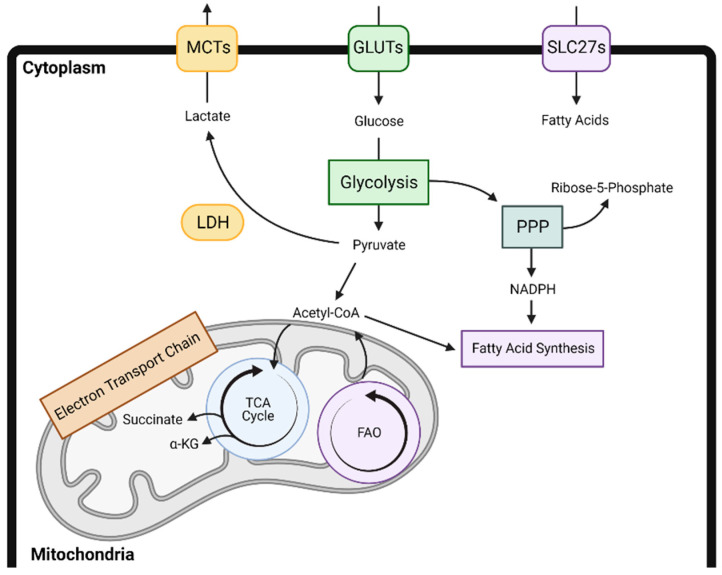
An overview of the major metabolic pathways including glycolysis, the tricarboxylic acid cycle, fatty acid synthesis, fatty acid oxidation, oxidative phosphorylation, and the pentose phosphate pathway and their interactions. α-KG—alpha-ketoglutarate; FAO—fatty acid oxidation; GLUT—glucose transporter; LDH—lactate dehydrogenase; MCT—monocarboxylate transporter; PPP—pentose phosphate pathway; SLC27—Solute carrier family 27; TCA—tricarboxylic acid.

Decades of research have demonstrated the role of metabolic adaptations for cancer cell survival, proliferation, and progression [1,2,3]. These alterations in glycolysis, mitochondrial respiration, and other metabolic programs also alter the local tumor microenvironment (TME), leading to a depletion of nutrients and induction of local acidification or hypoxia. A growing body of evidence suggests that immune cells depend on similar metabolic changes for their recruitment, proliferation, and effector function, thus ultimately influencing the outcome of antitumor immune responses [4,5,6]. For example, in a variety of immune cell subtypes, increased glycolysis leads to immune activation, whereas increases in fatty acid oxidation, oxidative phosphorylation, and lipid uptake contribute to immune suppression. However, these metabolic alterations and ultimate impact on the local tumor microenvironment are cell type and context dependent. A broad overview of the major cellular metabolic pathways is presented in Figure 1.

**Figure 2:**
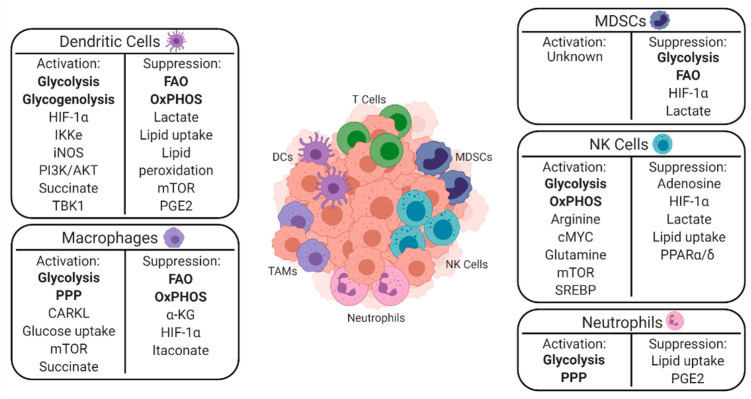
A depiction of innate immune cells within the TME and a summary of the metabolic pathways that lead them to promote either immune activation or suppression. Major metabolic pathways are denoted in bold, with key signaling molecules listed below them. CARKL—carbohydrate kinase-like protein; cMYC - HIF-1α—hypoxia inducible factor 1-alpha; IKKɛ - IkB kinase-ɛ; iNOS—inducible nitric oxide synthase; mTOR—mammalian target of rapamycin; OxPHOS—oxidative phosphorylation; PGE2—prostaglandin E2; PI3K—phosphatidylinositol 3-kinase; PPAR—peroxisome proliferator-activated receptor; SREBP1—sterol regulatory element binding protein; TBK1—tank binding kinase 1.

A greater understanding of the mechanisms underlying the interplay between cancer and immune cell metabolism is particularly important to understand given the recent advent of immunotherapies such as adoptive cell therapy and immune checkpoint blockade. Despite the success of these treatments, many patients do not respond, and others relapse after an initial period of response [7,8]. In several instances, metabolic changes within the TME drive these initial poor responses and influence the development of relapse [4,9]. Since T cells have received the most attention to date as the immediate effectors of most immunotherapies, this review aims to summarize current research related to the innate immune system, metabolism, and cancer [10,11,12]. This paper first discusses the specific metabolic requirements of innate immune cells for immune activation and suppression and summarizes them in Figure 2. This paper concludes by highlighting ongoing clinical applications of these findings.

## 2. Dendritic Cells

Dendritic cells (DCs) comprise a relatively small population in the tumor microenvironment but are essential for the initiation of antigen-specific immunity [13]. DCs receive and integrate environmental signals sensed by receptors for cytokines, damage-associated molecular patterns (DAMPs), and pathogen-associated molecular patterns (PAMPs). They then shape the immune response by processing and presenting antigens to T cells and modulating the activity of additional immune cells via cell–cell contacts and cytokine release [14]. Specific subsets of DCs include conventional DCs (cDCs), which play a crucial role in promoting antitumor CD4+ and CD8+ T cell responses, and plasmacytoid DCs (pDCs), which have been linked to immunosuppression and tolerance [15,16,17,18,19]. Plasticity amongst these populations is controlled by site-specific factors. Understanding how changes in metabolism alter the recruitment and behavior of DC subsets in the tumor microenvironment remains an important area of interest given the long-standing history of DC-based cancer vaccines and need to improve their therapeutic efficacy [20]. 

### 2.1. Immune Activation

Toll-like receptor (TLR) agonism triggers cDC activation and maturation and shifts their metabolism from oxidative phosphorylation to glycolysis to support their anabolic demands and allow for antigen presentation [21]. Within minutes of exposure to TLR agonists, phosphoinositide 3-kinase (PI3K)/protein kinase B (PKB/AKT), TANK-binding kinase 1 (TBK1), and IkB kinase-ɛ (IKKɛ) pathway signaling drives this metabolic switch to glycolysis, which can then be inhibited by adenosine monophosphate (AMP)-activated protein kinase (AMPK) or by the anti-inflammatory cytokine IL-10 [21,22]. The initial process driving the metabolic switch is inducible nitric oxide synthase (iNOS) independent and is directly controlled by the rate-limiting glycolytic enzyme hexokinase. However, sustained dendritic cell differentiation and maturation that occur following this depend on TLR-driven upregulation of iNOS for NO production to inhibit mitochondrial electron transport [22,23]. Notably, intracellular glycogen stores support this boost in glycolysis, and inhibition of glycogenolysis with the glycogen phosphorylase inhibitor CP91149 abrogates TLR-mediated DC activation and maturation [24]. It appears that hypoxia and hypoxia-inducible factor-1-α (HIF-1α) signaling complement this metabolic program. Increased HIF-1α protein levels accompany DC activation, and RNAi knockdown of HIF-1α diminishes glucose use by DCs, inhibits their maturation, and impairs their ability to stimulate T cells [25]. In addition to differentiation and maturation, glycolytic metabolism in DCs is essential for DC motility, chemokine receptor 7 (CCR7) oligomerization, and migration to draining lymph nodes [26]. Finally, metabolic pathway intermediates can also independently regulate DCs. For example, succinate, a TCA cycle intermediate, signals through the G protein-coupled receptor GPR91 and activates DCs to promote immune stimulation [27]. Mice deficient in this receptor suffer from impaired DC migration and diminished immune responses. 

**Figure 3:**
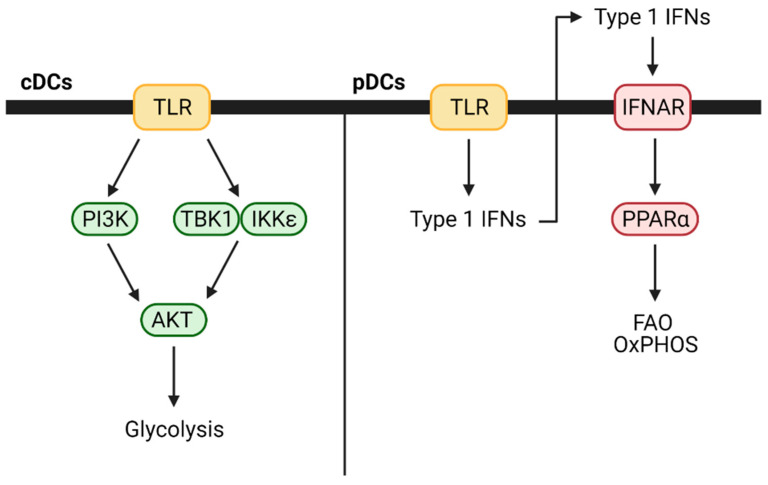
Metabolic changes that occur in DC subsets following TLR activation. IFNAR—interferon alpha-beta receptor; TLR—toll-like receptor.

In contrast to cDCs, when pDCs are activated with TLR7 or TLR9 agonists, they increase fatty acid oxidation and oxidative phosphorylation without any changes to glycolysis [28]. This likely occurs due to autocrine or paracrine type 1 interferon signaling as pDCs secrete interferon (IFN)-α and IFN-β following TLR stimulation and treatment with either is sufficient to increase fatty acid oxidation and oxidative phosphorylation. The process also depends on mitochondrial pyruvate import and fatty acid synthesis. Blocking either prevented TLR-based activation and metabolic changes. These differences between cDCs and pDCs are highlighted in Figure 3. DC subset-specific metabolic demands have not been extensively studied in mouse cancer models, but it is possible that they play a prominent role in modulating the overall antitumor immune response. 

### 2.2. Immune Suppression

Although TLR signaling induces glycolysis to activate DCs, and the presence of glucose in the tumor microenvironment has been classically thought to be important for DC activation of T cells, this relationship now appears more complex than previously thought. In a tissue- and context-dependent manner, glucose can also paradoxically repress DC inflammatory outputs and DC-induced T cell proliferation and effector function [29]. A feedback circuit integrating mechanistic target of rapamycin (mTOR), HIF1α, and iNOS senses local glucose, arginine, leucine, and NO and then orchestrates the metabolic and functional changes in TLR-stimulated DCs. Ultimately, glucose-deprived DCs show increased costimulatory molecule and IL-12 expression, both of which are key signals for T cell activation. mTOR inhibition with rapamycin replicates the effects of glucose deprivation and increases the proinflammatory outputs of DCs [30]. In fact, pretreatment of DCs with rapamycin before the application of a DC-based vaccine in the B16 melanoma model expanded their lifespan, increased expression of costimulatory molecules, and improved antigen-specific CD8+ T cell generation and antitumor immunity [31]. 

Cancer cells frequently reprogram lipid metabolism to meet their bioenergetic demands for the synthesis of membranes and signaling molecules, and these changes to lipid metabolism can also alter immune cell behavior [32]. For example, tumor-produced prostaglandin E2 (PGE2) impairs NK cell viability and chemokine production and reduces chemokine receptor expression by cDC1s [33]. This promotes tumor immune evasion as cDCs depend on the release of chemoattractants by NK cells for their recruitment to the tumor site. Excess lipid accumulation in DCs through upregulation of the scavenger A receptor (SRA) and macrophage scavenger receptor 1 (MSR1) also leads to DC dysfunction [34]. Cancer patients and tumor-bearing mice often have increased triglyceride levels. When these triglycerides enter DCs, they profoundly impair their ability to process and present soluble antigens without altering major histocompatibility complex (MHC) or costimulatory molecule expression. This appears to only impact cDCs, as mouse and human pDCs do not upregulate MSR1 or SRA and accumulate lipids. Importantly, blocking fatty acid synthesis with the acetyl-CoA carboxylase-α inhibitor TOFA in tumor-bearing mice prior to DC-based vaccine administration improved antitumor immune responses and may warrant further evaluation [34]. Additionally, DC metabolism can be influenced by intracellular lipids that undergo peroxidation, triggering the endoplasmic reticulum (ER) stress response factor XBP1 to disrupt DC homeostasis and blunt antitumor immunity [35]. Inhibiting XBP1 with siRNA boosted T cell activation and increased survival in mice with ovarian tumors. 

Peroxisome proliferator-activated receptors (PPARs) are fatty acid-activated transcription factors that regulate energy metabolism, and PPARγ is especially important for glucose lipid storage [36]. Paracrine signaling via the Wnt5α-β-catenin-PPAR-γ pathway in DCs can also establish a favorable immune context for tumor growth. Melanomas use Wnt5α to upregulate lipid uptake and carnitine palmitoyltransferase-1A (CPT1A) in DCs, driving fatty acid oxidation and oxidative phosphorylation, leading to the establishment of a site with immune privilege [37]. Again, inhibiting this process boosts antitumor immunity. 

Glucose and lipids are not the only factors that promote DC tolerization and immunosuppression. When lactic acid accumulates near DCs, it induces both early and long-lasting metabolic reprogramming. This reduces production of IL-12, IL-23, and tumor necrosis factor α (TNFα) and activation of T cells [38]. These findings have important implications for both the preparation of DC-based vaccines in culture and DC behavior within the tumor microenvironment. 

Adenosine signaling and its influence on dendritic cells has been well described following kidney ischemia reperfusion injury (IRI). In this model system, adenosine signaling induces a tolerogenic DC phenotype with reduced expression of costimulatory molecules and activation of NKT cells [39]. In this setting of IRI, reduced inflammation protects nearby tissue from damage, but a similar effect in the TME could facilitate tumor progression. Testing of adenosine signaling blockade with small molecule inhibitors is underway for several types of cancer, and it will be important to consider the effects of these treatments on DCs going forward [40].

## 3. Macrophages

Tumor-associated macrophages (TAMs) fall along a phenotypic spectrum ranging from both pro-tumor, anti-inflammatory “M1” phenotypes to antitumor, pro-inflammatory “M2” phenotypes and influence cancer cell survival, proliferation, and invasiveness in the TME by altering local immune cell behavior. These TAM subsets exhibit plasticity in response to changing signaling in the local microenvironment and are capable of being re-educated [41]. For example, in a mouse model of pancreatic adenocarcinoma, tumor-associated macrophages induce both immunosuppression of T cells via IL-6 and IL-10 as well as desmoplasia and fibrosis via platelet-derived growth factor [42]. This pro-tumor, anti-inflammatory phenotype also predominates in pancreatic cancer patients, with macrophage infiltration often correlating with higher tumor grade, progression, and increased rates of recurrence [43,44]. Tumor-associated macrophages are not reprogrammed in response to immune checkpoint inhibition and therefore represent a major barrier to increase efficacy for these therapies [45]. As strategies that attempt to manipulate macrophage polarization to potentiate immunotherapies and other treatment regimens are investigated, components of the metabolic pathways that regulate this process should be considered as potential targets. 

### 3.1. Immune Activation

Glucose metabolism is a key driver of macrophage inflammatory capacity and is dependent on the pentose phosphate pathway (PPP) generation of NADPH for ROS production and subsequent oxidative burst, allowing for effector activity of these cells. Glycolytic genes such as GLUT1 become overexpressed after stimulation with TLRs in macrophages which leads to an increase in ROS and proinflammatory mediators [46]. A natural increase in GLUT1 has also been observed in macrophage-induced tissue inflammation in mice fed a high-fat diet, and inhibiting glycolysis or treating cells with the antioxidant N-acetylcysteine reduces this effect. It is hypothesized that obesity leads to a ‘meta-inflammatory’ state in patients. For example, in triple-negative breast cancer (TNBC), obese patients respond better to PD-L1 immune checkpoint blockade but are also at greater risk of developing immune-related adverse events (irAEs) [47]. Similar results have been reported in melanoma, non-small-cell lung cancer, and renal cell carcinoma [48]. Other stressors such as alcohol exposure, burn, and sepsis also upregulate GLUT1 expression on macrophages [49,50]. However, due to the relative acuteness of these stressors, they are less likely to alter immunotherapy outcomes. 

mTOR signaling can enhance glycolysis in macrophages to promote inflammation via the NLRP3 inflammasome in a hexokinase-1 (HK1)-dependent manner [51]. Glucose deprivation and genetic or pharmacologic inhibition of mTOR signaling or HK1 in macrophages all blunted glycolysis and prevented NLRP3 inflammasome-mediated caspase-1 activation. In alveolar macrophages, the mTOR complex component raptor has also been linked to macrophage proliferation and phagocytic ability, but this remains to be shown in other contexts [52]. Finally, the sedoheptulose kinase carbohydrate kinase-like protein (CARKL) modulates glycolytic energy flux to define macrophage activation and polarity [53]. Under normal conditions, CARKL inhibits glycolysis in macrophages. However, when they are exposed to LPS or other activators, rapid downregulation of CARKL permits an increase in glycolysis, whereas overexpression of CARKL completely prevents macrophage proinflammatory polarization in response to these activators. CARKL therefore is an important mediator in the balance of M1 or M2 polarization and could be targeted with agents such as rapamycin. 

### 3.2. Immune Suppression

Increased oxidative phosphorylation reduces glycolysis and promotes the immunosuppressive polarization of macrophages. In response to IL-4, signal transducer and activator of transcription 6 (STAT6) and PPARgamma-coactivator-1-beta (PGC-1β) upregulate macrophage fatty acid oxidation and mitochondrial biogenesis [54]. In turn, they strongly downregulate proinflammatory cytokine production, which can be reversed with RNAi knockdown of PGC-1β. Anti-inflammatory cytokines such as IL-10 and TGFβ also direct macrophages toward an immunosuppressive phenotype via AMPK signaling [55]. AMPK is rapidly phosphorylated which triggers reduced IκB degradation, enhanced AKT activity, glycogen synthase kinase 3 β (GSK3-β) inhibition, and cyclic AMP response element-binding protein (CREB) activation. This metabolic switch is also bidirectional: when macrophages encounter pro-inflammatory stimuli, AMPK is dephosphorylated and inactivated and reverse polarization occurs. Whether these changes alter other metabolic pathways has not yet been studied.

Several TCA cycle intermediates also modulate the proinflammatory state of macrophages. On its own, succinate acts as an inflammatory signal that stabilizes HIF-1α and induces IL-1β expression [56]. Mitochondrial oxidation of succinate via succinate dehydrogenase (SDH) induces a pro-inflammatory gene expression profile, and blocking succinate oxidation with dimethyl malonate (DMM) inhibits this inflammatory phenotype [57]. SDH can be endogenously inhibited by itaconate and its regulator, immunoresponsive gene 1 (IRG1), to encourage succinate accumulation [58,59]. Itaconate is produced from aconitate, another TCA cycle intermediate, in macrophages activated with TLR ligands or interferons [60,61]. In mouse models of melanoma and ovarian carcinoma, accumulation of itaconate increases mitochondrial oxidative phosphorylation and ROS production and potentiates tumor growth while lentiviral knockdown of IRG1 reduces it [62]. Lastly, alpha-ketoglutarate (α-KG) prevents pro-inflammatory polarization of macrophages by suppressing IKKβ-NF-κβ pathway activation [63]. This occurs by the prolyl hydroxylase (PHD)-dependent proline hydroxylation of IKKβ. 

Hypoxia is another critical factor in macrophage polarization. First, hypoxia-induced semaphorin 3A (Sema3A) and vascular endothelial growth factor (VEGF) act as chemoattractants for macrophages to the tumor microenvironment [64]. Once there, they acquire a pro-angiogenic and immunosuppressive phenotype due to the tumor-derived cytokines Oncostatin M and Eotaxin [65]. Blockade of Oncostatin M and Eotaxin prevented both macrophage recruitment to and anti-inflammatory polarization in the microenvironment of breast cancer cells and slowed tumor progression. Interestingly, this treatment also enhanced the anti-angiogenic efficacy of the VEGF inhibitor bevacizumab. In mouse models of lung adenocarcinoma and melanoma, tumor-derived lactate pushes macrophages toward an anti-inflammatory phenotype and promotes tumor growth in a HIF-1α-dependent manner [66]. Interestingly, pyruvate kinase M2 (PKM2) and HIF-1α can bind directly to hypoxia response element (HRE) sites on the PD-L1 (programmed death-ligand 1) promoter to upregulate PD-L1 expression by macrophages and promote tumor growth by restraining CD8+ T cell responses [67]. When pyruvate metabolism in macrophages is restricted by V-set immunoglobulin-domain-containing 4 (VSIG4)-induced PI3K/AKT/STAT3 signaling, mitochondrial oxidation decreases and they develop a pro-inflammatory phenotype [68]. 

**Figure 4:**
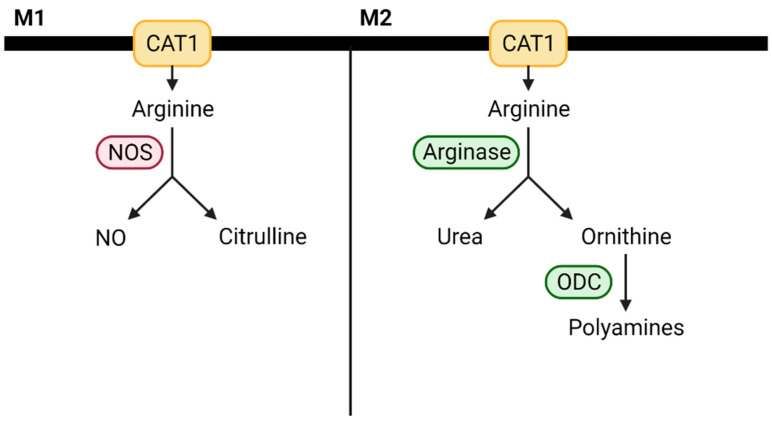
Alterations in arginine metabolism influence macrophage polarizaiton. CAT1—cationic amino acid transporter 1; NO—nitric oxide; ODC—ornithine decarboxylase.

Amino acids and their derivatives also modify macrophage activity. Arginine metabolism, for example, has been identified as a key driver of macrophage polarization [69]. Macrophages can metabolize arginine into NO, a proinflammatory mediator, and citrulline via NOS or hydrolyze arginine into ornithine and urea via arginase [70]. These processes directly compete, and M1 macrophages tend to have increased NOS activity, whereas M2 macrophages have elevated arginase expression. These differences are shown in Figure 4. It is important to note that depending on the specific context, both M1 and M2 macrophages can be proinflammatory or anti-inflammatory, and the impact of macrophage arginine metabolism has not yet been studied in the context of cancer. 

Immunosuppressive polarization of macrophages has also been linked to increased glutamine catabolism and UDP-GlcNAc biosynthesis, and glutamine deprivation or inhibition of N-glycosylation blocks this phenotype [71]. Inhibition of glutamine synthetase also decreases intracellular glutamine and skews macrophages toward a pro-inflammatory state where they possess increased succinate levels and enhanced glycolysis [72]. Finally, adenosine enhances the immunosuppressive polarization of macrophages in response to cytokines such as IL-4 and IL-13 through A2A and A2B receptor signaling [73]. Furthermore, adenosine signaling can actively suppress the TLR-dependent expression of inflammatory cytokines such as IL-12, IFNγ, and TNF-α independently from IL-4 signaling [74]. Notably, adenosine is produced under hypoxic conditions and therefore likely influences macrophage behavior in the tumor microenvironment. 

## 4. MDSCs and Neutrophils

Myeloid cells are classically activated by strong signals in the form of PAMPs and DAMPs which cause them to terminally differentiate into DCs and macrophages. However, persistent stimulation with chronic, low strength signals in settings of prolonged infection or cancer pathologically transforms myeloid cells into myeloid-derived suppressor cells (MDSCs) with distinct biochemical profiles and functional activity [75]. Two primary subtypes of MDSCs exist: monocytic, mononuclear MDSCs (M-MDSCs) and granulocytic, polymorphonuclear MDSCs (PMN-MDSCs). Across multiple cancer types, total MDSCs in peripheral blood positively correlates with cancer stage and tumor burden and negatively correlates with therapy response and overall survival [76]. They have also been implicated in the creation of pre-metastatic niches for spread of cancers, notably pancreatic adenocarcinoma [77]. Therapeutic approaches that attempt to deplete MDSCs with chemotherapeutic agents and tyrosine kinase inhibitors, block their activation or recruitment with CCL2 and CCR5 blockade, or inhibit their immunosuppressive functions with targeted STAT3 inhibitors are all under study [78]. More recently, efforts to target MDSCs have also included targeting metabolic pathways including fatty acid oxidation [79]. 

Apart from their ability to differentiate into PMN-MDSCs, neutrophils can influence cancer progression in a variety of ways. [80]. Multiple clinical studies have identified an association between an elevated neutrophil-lymphocyte ratio (NLR) and a poor treatment response and overall survival rate in many solid tumors [81]. While the action of PMN-MDSCs partly accounts for this, tumor-associated neutrophils (TANs) also play a role. At the tumor site, pro-tumor TANs reduce T cell proliferation and IFNγ production and induce T cell apoptosis to suppress T cell-mediated immunity [82]. They can also facilitate metastasis by promoting angiogenesis and enhancing dissemination and distant seeding through release of matrix metalloproteinase-9 (MMP-9) [83]. Equally important are neutrophil-derived extracellular DNA (NETs) which have been implicated in the pro-tumor functions of neutrophils and found at increased levels in the plasma of cancer patients [84,85].

Although studies have mainly focused on the role in pro-tumor progression of TANs, antitumor effects have also been described. TANs alter macrophage polarization, recruit and activate CD4+ and CD8+ T cells, promote tumor cell sloughing from basement membranes, induce direct tumor cell apoptosis by secreting cytotoxic ROS, and can even elicit antibody-dependent cell cytotoxicity [86]. Given these varied functions of neutrophils throughout tumorigenesis, understanding how metabolism modulates neutrophil behavior in the tumor microenvironment is critical. 

### 4.1. Immune Activation

MDSCs rarely contribute to immune activation. TANs, however, appear to rely primarily on glycolysis for their bioenergetic needs rather than oxidative phosphorylation which is in contrast to other immune cells. Chacko et al. isolated circulating lymphocytes, neutrophils, monocytes, and platelets from healthy donors and evaluated their baseline oxygen consumption rate (OCR) and response to mitochondrial inhibitors [87]. These experiments showed that neutrophils were almost entirely unresponsive to mitochondrial inhibitors, possess relatively few mitochondria compared to other immune cells, and operate near full glycolytic capacity at baseline. Metabolic sensors such as prolyl hydroxylase regulate glycolytic flux to ensure proper neutrophil motility, functional capacity, and survival [88]. 

Neutrophil NET formation is also dependent on glucose-driven glycolysis and to a lesser extent glutamine [89]. Neutrophil stimulation with PMA upregulated GLUT1 and increased glucose uptake and lactate production. NET formation did not occur in the presence of glucose-free culture media or after treatment with the glycolysis inhibitor 2-deoxy-glucose (DOG), and it was markedly diminished in the absence of glutamine. The pentose phosphate pathway and, in particular, glucose-6-phosphate dehydrogenase (G6PD) also appear to supplement NET formation. Like GLUT1, G6PD activity increases upon neutrophil stimulation, and when G6PD is blocked by the NADPH inhibitor 6-aminonicotinamide (6-AN), NET formation is reduced [90]. It is speculated that NADPH produced by the PPP is necessary for NADPH oxidase (NOX)-dependent ROS generation and subsequent NET release. Interestingly, immature low-density neutrophils (iLDNs), known to promote liver metastasis through both NETs and angiogenesis induction, exhibit a higher spare glycolytic capacity and greater ability to engage in oxidative phosphorylation than their high-density counterparts [91]. This metabolic flexibility allows them to function normally even in glucose starved conditions.

### 4.2. Immune Suppression

When MDSCs encounter tumor cells, they upregulate several glycolytic enzymes [92]. Upregulation of glycolysis prevents the accumulation of excess ROS through the glycolytic intermediate and antioxidant phosphoenolpyruvate (PEP), protects them from apoptosis, and promotes suppression of T cell activity via arginase, iNOS, PD-L1, and PD-L2. Inhibition of glycolysis with 2-DG elicits MDSC apoptosis, reduces their overall presence at tumors, and improves antitumor immune responses, and treatment with PEP alone was sufficient to protect MDSCs from apoptosis and block this effect [92]. Increased tumor glycolysis also supports MDSC development through the production of granulocyte colony-stimulating factor (G-CSF) and granulocyte macrophage colony-stimulating factor (GM-CSF) [93]. The CCAAT/enhancer-binding protein beta (CEBPB) isoform liver-enriched activated protein (LAP) controls tumor G-CSF and GM-CSF production. Restricting tumor glycolysis depletes ATP relative to AMP and activates the energy sensing AMPK-ULK1-autophagy pathway which then modulates levels of LAP. This ultimately limits MDSC development and improves antitumor immunity. Tumor glycolysis also increases MDSC development through lactate production [94]. Knockdown of LDH-A in pancreatic cancer cells injected into mice resulted in a lower frequency of MDSCs and smaller tumors, and placing mice on a ketogenic diet to reduce glucose and lower lactate production replicated this effect. In addition to glycolysis, tumor-associated MDSCs upregulate fatty acid uptake and oxidation which leads to elevated arginase and NO production and increased ability to inhibit T cell proliferation [79]. Blocking fatty acid oxidation with etomoxir prevented the tolerogenic functions of MDSCs, resulted in T cell-dependent inhibition of tumor growth, and enhanced the antitumor effect of low-dose chemotherapy and adoptive T cell therapy. 

Other metabolic pathways also influence MDSC activity. Inhibition of ornithine decarboxylase (ODC), which decarboxylates the urea cycle product ornithine to initiate polyamine synthesis, impaired MDSC function and improved T cell-dependent antitumor immunity by reducing MDSC arginase and CD39/CD73 expression [95]. L-ornithine has previously been shown to inhibit arginase, and the therapeutic potential of this metabolic axis requires deeper examination [96]. Hypoxia-driven HIF-1α expression is also associated with elevated arginase and iNOS expression and increased T cell suppression by MDSCs [97]. Either exposing spleen MDSCs to hypoxia or directly transferring them to the tumor microenvironment was sufficient to replicate this tumor MDSC phenotype [97]. HIF-1α also directly binds to a transcriptionally active HRE in the PD-L1 promoter to induce rapid and dramatic upregulation of PD-L1 by MDSCs [98]. Consequently, PD-L1 blockade in the setting of hypoxia leads to downregulation of IL-6 and IL-10 by MDSCs and improved T cell activation. 

Other studies have focused on the metabolic shifts that specifically contribute to the development of PMN-MDSCs from neutrophils. Patel et al. describe this as a two-step process [99]. First, bone marrow-derived neutrophils upregulate glucose metabolism, ATP production, and migratory ability but otherwise maintain their typical function. However, following accumulation at tumor sites, they acquire an immunosuppressive capacity. This was confirmed in tumor-bearing mice and in the blood of cancer patients. Rice et al. further identified tumor-derived c-Kit ligand (SCF) as a driver of c-Kit maintenance in neutrophils, which supports increased mitochondrial mass, function, and fatty acid oxidation and enables ROS generation and T cell suppression [100]. Additional neutrophil reprogramming in the tumor microenvironment occurs through fatty acid transport protein 2 (FATP2) [101]. FATP2 upregulation dramatically increases intracellular lipids such as arachidonic acid in neutrophils isolated from tumors compared to those from spleens. Neutrophils can then synthesize PGE2 from arachidonic acid to suppress antitumor immune responses. Importantly, pharmacological inhibition of FATP2 delays tumor progression and even synergizes with immune checkpoint blockade. PGE2 is not the only molecule released by neutrophils in the tumor microenvironment to elicit immunosuppression. When triggered by IL-8 and TNFα, they will also unleash arginase-filled granules that catabolize extracellular arginine and inhibit T cell proliferation as a result [102]. Multiple preclinical studies have since evaluated PGE2 and arginase as therapeutic targets either alone or in combination with checkpoint inhibitors [103,104]. Inhibition of arginase by CB1158 blocks myeloid cell-mediated suppression of T cell proliferation in vitro and reduced tumor growth in multiple cancer models, as both a single agent and in combination with checkpoint blockade, adoptive T cell transfer, adoptive NK cell transfer, and the chemotherapeutic agent gemcitabine [105]. A recent phase 1 clinical trial tested CV1158 as a monotherapy and in combination with the PD-1 inhibitor pembrolizumab in both naïve and checkpoint inhibitory refractory advanced and metastatic solid tumors. Early results indicate that the combination is well tolerated and showed responses in both groups [106]. 

## 5. NK Cells

Natural killer (NK) cells are cytotoxic large granular lymphocytes (LGLs) that identify target cells and induce antibody mediated cellular cytotoxicity (ADCC) or apoptosis by releasing cytolytic granules. Tumor cells often diminish their expression of major histocompatibility complex (MHC) class 1 molecules or overexpress NK cell-activating receptor ligands and trigger their own destruction by NK cells [107]. For patients with many solid tumors, expression levels of NK-cell activating receptors and NK cell infiltration into tumors can predict overall survival and treatment response [108]. NK cells are also critical for tumor immunosurveillance and potentiate inflammatory responses through local secretion of cytokines and chemokines. 

The recent advent and success of immune checkpoint blockade and chimeric antigen receptor (CAR) T cell immunotherapies has raised considerable interest in the therapeutic potential of NK cell-based therapies. In multiple pathogen infections and cancers, activated NK cells have been shown to express the immune checkpoints programmed cell death protein 1 (PD1), cytotoxic T lymphocyte-associated antigen 4 (CTLA4), and T cell immunoreceptor with Ig and ITIM domains (TIGIT) [109,110,111,112,113]. Furthermore, NK cells are often indispensable to the successful antitumor effect of checkpoint blockade, and NK cell-specific checkpoint targets such as NKG2A have proven effective in mouse models [114,115]. The clinical applications of CAR T strategies are often limited by side effects such as cytokine release syndrome (CRS) caused by overstimulation of the immune system. CAR NK alternatives might avoid this because NK cells have a shorter lifespan and do not secrete autocrine growth factors such as IL-2 [116]. Unlike CAR T cells, even if NK cell targeted antigens are lost from tumors, CAR NK cells will still recognize their endogenous activating receptors and should be influenced less by inhibitory receptor expression. Many of the studies discussed below have powerful implications for the adoptive transfer of in vitro-activated or genetically engineered NK cells. 

### 5.1. Immune Activation

The metabolic checkpoint mTOR is one of the primary regulators of NK cell development and effector function. NK cells progress to quiescence as they mature unless they are stimulated by the cytokines IL-15, IL-18, or IL-12 [117,118]. Under these conditions, mTOR promotes either NK cell proliferation in the bone marrow or activation in the periphery where they upregulate glucose uptake, glycolysis, oxidative phosphorylation, and IFNγ and granzyme B expression [118,119]. Limiting glycolysis with 2-DG or inhibiting mTOR with rapamycin abrogates NK cell cytotoxicity in both mice and humans and leads to immunosuppression [117,119]. Transforming growth factor-β (TGF-β) signaling also inhibits mTOR activity and arrests NK cell development by blocking its activation by IL-15 [120]. Interestingly, deletion of a TGF-β receptor subunit enhanced NK cell effector function and limited metastasis in mouse models of melanoma and lung cancer. Indirect factors that inhibit mTOR similarly inhibit NK cells. In a mouse model of diet-induced obesity, peroxisome proliferator-activated receptor α/δ (PPARα/δ) drove lipid accumulation within NK cells, inhibited mTOR signaling, reduced glucose uptake, glycolysis, and oxidative phosphorylation, and impaired NK cell effector function [121]. Treatment with exogenous fatty acids or PPARα/δ agonists replicated this, and inhibiting PPARα/δ or blocking lipid uptake into NK cell mitochondria reversed it. Finally, mTOR regulates glycolysis and lipid synthesis through the transcription factors cMYC and sterol regulatory element-binding protein (SREBP) respectively, and both of these dictate NK cell function in response to cytokine stimulation. cMYC is necessary for cytokine-induced stimulation, glucose uptake, glycolysis, and oxidative phosphorylation in NK cells and their subsequent growth, proliferation, and effector function [122]. Furthermore, glutamine transport through the amino acid transporter SLC7A5 tightly regulates cMYC expression, and inhibition of L-amino acid transport also impairs metabolic and functional responses to cytokines by NK cells. Glutamine metabolism via glutaminolysis, however, does not impact these processes. Like cMYC, SREBP is required for cytokine-induced metabolic reprogramming and activation of NK cells [123]. Interestingly, this occurs independently from its regulation of lipid synthesis as inhibition of acetyl-CoA carboxylase and fatty acid synthase did not alter glycolysis and oxidative phosphorylation. SREBP instead directs metabolized glucose toward the citrate-malate shuttle, and direct inhibition of the shuttle replicates the effect of SREBP loss. Notably, multiple endogenous SREBP inhibitors such as 27-hydroxycholesterol exist within the tumor microenvironment and could contribute to immunosuppression by blunting NK cell effector function [124,125,126]. 

### 5.2. Immune Suppression

Given the importance of glycolysis and oxidative phosphorylation for NK cell development and effector function, processes that manipulate these pathways can suppress the antitumor activity of NK cells. In a KRAS-driven mouse model of lung cancer, aberrantly elevated fructose-1,6-bisphosphatase (FBP1) expression in NK cells from the tumor microenvironment impaired their function by inhibiting glycolysis and reducing their proliferation and viability [127]. FBP1 is the rate-limiting enzyme for gluconeogenesis and inhibits glycolysis, and inhibition of FBP1 restored the function of tumor-associated NK cells. Furthermore, pretreatment of adoptively transferred NK cells with an FBP1 inhibitor dramatically enhanced their capacity to slow tumor growth and suggests a potential direction for NK cell-based immunotherapy methods. 

Elevated lactate dehydrogenase A (LDHA) is associated with poor outcomes in human cancer patients, and tumor secreted lactate impairs NK cell function in several mouse tumor models. Pretreatment of both mouse and human NK cells with lactate inhibits their cytolytic ability and decreases NKp46 expression [94]. NKp46 participates in tumor cell killing by recognizing receptor-specific ligands. In both melanoma and pancreatic adenocarcinoma cell lines, knockdown of LDHA resulted in increased NK cell proliferation, IFNγ and granzyme B production, and tumor control by immune cells [128]. In a model of liver metastases, NK cells treated with tumor-conditioned media in vitro showed signs of mitochondrial stress and even underwent apoptosis due to elevated lactate [129]. Intracellular acidification due to lactate accumulation disturbs nuclear factor of activated T cells (NFAT) production in NK cells, and this could explain the observed suppression of IFNγ expression and why neutralizing tumor acidity improves immunotherapy responses [128,130]. 

Elevated lactate levels, aberrant mTOR signaling, and the hypoxic conditions of the tumor microenvironment can all activate HIF-1α, which has been shown to impair NK cell effector function in a variety of settings. When NK cells upregulate HIF-1α in response to tumor hypoxia, they fail to increase their expression of major activating surface receptors NKp46, NKp30, NKp44, and NKG2D and degranulate in response to cytokines [131]. Despite this, NK cells retain their ability to kill tumor cells via ADCC as hypoxia does not alter their expression of CD16, the Fc-γ receptor. Paradoxically, deletion of HIF-1α in NK cells reduces tumor growth in mice despite blunted NK cell tumor killing [132]. This occurs because NK cells are an essential source of the soluble form of VEGF receptor 1 (sVEGFR1), an angiostatic ligand that negatively regulates VEGF bioavailability in the tumor environment and impairs tumor nutrient acquisition. 

Similar to how glutamine uptake regulates cMYC control of NK cell effector function, availability of amino acids beyond glutamine can also influence NK cell function. Lowering the arginine availability to mouse and human NK cell lines impairs their proliferation, viability, and cytotoxicity. This also reduces their expression of NKp46 and NKp30 and decreases IFNγ production without altering genes for arginine uptake or metabolism [133]. Consequently, PMNs that synthesize the arginine-hydrolyzing enzyme arginase can accumulate and locally deplete arginine to suppress NK cell proliferation and IFNγ secretion [134]. It is possible that nitric oxide (NO) synthases and other enzymes that metabolize arginine could also accomplish this, but this has not been confirmed. Finally, adenosine also acts as a key immunosuppressive metabolite that limits the maturation and proliferation of NK cells [135]. Adenosine signaling via the A2A adenosine receptor (A2AR) blocks effective antitumor immune responses partly by limiting NK cell infiltration into tumor sites. Treatment of mice with exogenous adenosine reduces the amount of mature NK cells that they produce, and deletion of A2AR or A2AR antagonism enhances NK cell maturation. Genetically or therapeutically targeting A2AR could prove vital for NK cell-based cancer immunotherapies. 

## 6. Clinical Applications—Opportunities and Challenges

Many widely used chemotherapies such as 5-fluorouracil, methotrexate, and gemcitabine target cancer cell metabolism to induce cell death. While these anti-metabolite chemotherapies have proven to be the cornerstone of many current regimens, the recent success of immunotherapy and our broadening understanding of immunometabolism is driving the study and rapid development of immunometabolism directed agents. The ideal agent would selectively target metabolic processes enriched in the tumor microenvironment as compared to agents that target conserved metabolic pathways which can lead to off target toxicities. [136]. However, there are a number of challenges associated with translating preclinical findings into effective clinical agents. First, immunometabolism is often studied in in vitro culture conditions which do not accurately reflect the dynamic biochemical and physical conditions of the TME [137,138]. Second, most in vivo studies of cancer immunometabolism have been carried out in mice, and it is unclear to what degree the metabolic pathways that govern mouse immune cell behavior overlap with humans [139]. Additionally, the time it takes to sort immune cell subsets after isolation can also result in changes to their metabolite pools [140]. Finally, when ex vivo samples such as plasma and serum are used for preclinical or clinical studies, the accuracy of the data extracted from these samples depends heavily on the preparation method used due to the rapid turnover of cellular metabolites that can occur prior to metabolism quenching [141]. 

Several ongoing clinical trials focus on combining metabolism-targeted agents with immunotherapy treatments. These are summarized in Table 1. Many of these trials exploit the use of previously approved drugs such as metformin and rosiglitazone which are used in the treatment of diabetes and alter downstream metabolic pathways. For example, metformin decreases peripheral insulin resistance by inhibiting mitochondrial respiration and activating AMPK. Activated AMPK inhibits metabolic processes such as gluconeogenesis and lipogenesis and stimulates glucose uptake and fatty acid oxidation thus affecting additional pathways tied in with immunometabolism [142]. Metformin can also target mTOR, insulin-like growth factor, and mitogen-activated protein kinase (MAPK) pathways [143]. In various preclinical studies, metformin has been shown to potentiate antitumor immunity more directly by promoting STING and Hippo signaling, PD-L1 degradation, and a reduction in tumor hypoxia [144,145,146,147]. Rosiglitazone activates PPARγ and has had similar preclinical results [148,149]. 

Novel compounds that specifically target immunometabolism are also undergoing testing. The arginase inhibitor CB1158 was discussed previously and is involved in multiple current trials. Telaglenastat is a glutaminase inhibitor that reduces the conversion of glutamine to α-KG and enhances the action of both anti-PD-1 and anti-CTLA-4 in mouse models of melanoma [150]. Indoleamine 2,3-dioxygenase (IDO1) catalyzes the conversion of tryptophan, an essential amino acid for protein synthesis and cell survival, to the immunosuppressive metabolite kynurenine. Despite the promise that IDO1 inhibitors showed in preclinical studies, the addition of the IDO1 inhibitor epacadostat to anti-PD-1 immunotherapy regimens failed to improve survival outcomes compared anti-PD-1 monotherapy [151]. 

Advances in metabolomics and additional new technologies will undoubtedly contribute to future immunometabolism-targeted drug discovery and development. For example, the combination of spatial metabolite profiling and high-dimensional immune cell imaging techniques will greatly improve our understanding of cell–cell metabolic interactions [152]. Single-cell energetic metabolism by profiling translation (SCENITH) is a recently published method for the complex metabolic profiling of samples ex vivo at single-cell resolution [153]. This approach overcomes many of the previously discussed challenges to using ex vivo samples and can help evaluate therapeutic responses and stratify patient populations. Additionally, established procedures such as the liquid and gas chromatography–mass spectrometry (LC–MS and GC–MS), nuclear magnetic resonance (NMR) spectrometry, and the Seahorse assay continue to improve.

Gong et al. recently generated a large multi-omics database to classify TNBC samples into three distinct metabolic categories and identify specific metabolic vulnerabilities that enhance immunotherapy responses [154]. Others are creating datasets which integrate metabolic characterization such as this with transcriptomics which will also help with developing new targets for therapy. Since there is a great deal of interindividual variability in how patients with the same cancer respond to treatments, accelerated advances in these approaches also have potential applications for personalized medicine.

## 7. Conclusions

Foundational insights into cancer cell metabolism pave the way for the interrogation of the importance of immunometabolism in the cancer setting. It is now clear that immune cells have specific metabolic dependencies that regulate their polarization and effector function, and targeting these pathways has the potential to improve cancer immunotherapy. While many existing approved drugs are undergoing testing in combination with immune checkpoint blockade and other immunotherapy agents, drugs currently in development that aim to target cancer and immune cell metabolism must consider the competing effects that they could exert on distinct cell populations. Genetically or pharmacologically altering key metabolic checkpoints ex vivo could also improve the efficacy of adoptive cell transfer therapies. Promising therapies including 2-deoxy-D-glucose (2DG) and AKT inhibitors, which when given systemically for cancer treatment were limited by toxicity, may be better utilized in ACT treatment [155,156]. In the future, it will be essential to understand how specific cell–cell interactions drive metabolic changes and determine the context specificity of individual immunometabolism pathways. Improvements in metabolomics technologies will inevitably propel our progress toward these goals. 

## Figures and Tables

**Table 1:** A summary of ongoing immunometabolism-targeted clinical trials. ECAR—extracellular acidification rate; KEAP1—kelch-like ECH-associated protein 1; LKB1—serine/threonine kinase 11; NRF2—nuclear factor erythroid 2; OCR—oxygen consumption rate; PD-1—programmed cell death protein 1; TAM—tumor-associated macrophage.

Drug	Mechanism of Action	Clinical Trials
Bicanorm	Sodium bicarbonate	Analysis of T cell metabolism (ECAR, OCR, cytokine production) in relapsed acute myeloid leukemia patients receiving donor lymphocyte infusions (NCT04321161)
INCB001158	Arginase inhibitor	Phase 1/2 study of INCB001158 alone or in combination with anti-PD-1 for patients with advanced/metastatic solid tumors (NCT02903914) [104] Phase 1/2 study of INCB001158 in combination with chemotherapy in subjects with solid tumors (NCT03314935)
Metformin	AMPK activation; mitochondrial glycerophosphate dehydrogenase inactivation	Anti-PD-1 plus/minus metformin in advanced melanoma, renal cell carcinoma, non-small-cell lung carcinoma, hepatocellular carcinoma, urothelial cancer, or head and neck squamous cell carcinoma (NCT04114136) Platinum chemotherapy and metformin plus/minus fasting mimicking diet to target the metabolic vulnerabilities of LKB1-inactive lung adenocarcinoma (NCT03709147) Anti-PD-1 with or without metformin in treating participants with head and neck squamous cell carcinoma to evaluate alterations in T cell and TAM polarization (NCT03618654)
Rosiglitazone	PPARγ agonist	Anti-PD-1 plus/minus rosiglitazone in advanced melanoma, renal cell carcinoma, non-small-cell lung carcinoma, hepatocellular carcinoma, urothelial cancer, or head and neck squamous cell carcinoma (NCT04114136)
Telaglenastat	Glutaminase inhibitor	KEAPSAKE: A study of telaglenastat with standard-of-care chemoimmunotherapy in 1L KEAP1/NRF2-mutated, non-squamous non-small-cell lung carcinoma (NCT04265534)

## Abbreviations

6-AN	6-aminonicotinamide
ADCC	Antibody-dependent cellular cytotoxicity
α-KG	α-ketoglutarate
AKT	Protein kinase B
AMP	Adenosine monophosphate
AMPK	AMP-activated protein kinase
CAR	Chimeric antigen receptor
CARKL	Carbohydrate kinase-like protein
CAT1	Cationic amino acid transporter 1
CCR7	C-C chemokine receptor type 7
cDC	Conventional dendritic cells
CEBPB	CCAAT/enhancer-binding protein beta
CPT1A	Carnitine palmitoyltransferase-1A
CREB	Cyclic AMP response element-binding protein
CRS	Cytokine release syndrome
CTLA4	Cytotoxic T lymphocyte-associated antigen 4
DAMP	Danger-associated molecular pattern
DC	Dendritic cell
DMM	Dimethyl malonate
DOG	2-deoxy-glucose
ECAR	Extracellular acidification rate
ER	Endoplasmic reticulum
FAO	Fatty acid oxidation
FATP2	Fatty acid transport protein 2
FBP1	Fructose-1,6-bisphosphatase
G-CSF	Granulocyte colony-stimulating factor
GC–MS	Gas chromatography–mass spectrometry
GLUT	Glucose transporter
GM-CSF	Granulocyte macrophage colony-stimulating factor
GSK3β	Glycogen synthase kinase 3β
G6PD	Glucose-6-phosphate dehydrogenase
HIF-1α	Hypoxia-inducible factor-1α
HK1	Hexokinase 1
HRE	Hypoxia response element
IFN	Interferon
IFNAR	Interferon-alpha/beta receptor
IL	Interleukin
iLDNs	Immature low-density neutrophils
IKKɛ	IkB kinase-ɛ
iNOS	Inducible nitric oxide synthase
iRAE	Immune-related adverse events
IRG1	Immunoresponsive gene 1
IRI	Ischemia reperfusion injury
KEAP1	Kelch-like ECH-associated protein 1
LAP	Liver-enriched activated protein
LC–MS	Liquid chromatography–mass spectrometry
LDHA	Lactate dehydrogenase A
LGL	Large granular lymphocyte
LKB1	Serine/threonine kinase 11
LPS	Lipopolysaccharide
MCT	Monocarboxylate transporter
M-MDSC	Mononuclear myeloid-derived suppressor cell
MDSC	Myeloid-derived suppressor cell
MHC	Major histocompatibility complex
MMP-9	matrix metalloproteinase-9
MSR1	Macrophage scavenger receptor 1
mTOR	Mechanistic target of rapamycin
NET	Neutrophil extracellular trap
NK	Natural killer
NLR	Neutrophil lymphocyte ratio
NMR	Nuclear magnetic resonance
NO	Nitric oxide
NOX	NADPH oxidase
NRF2	Nuclear erythroid factor 2
OCR	Oxygen consumption rate
ODC	Ornithine decarboxylase
OxPHOS	Oxidative phosphorylation
PAMP	Pathogen-associated molecular pattern
PD1	Programmed cell death protein 1
PD-L1	Programmed death-ligand 1
PD-L2	Programmed death-ligand 2
pDC	Plasmacytoid dendritic cells
PEP	Phosphoenolpyruvate
PGC-1β	PPARgamma-coactivator-1β
PGE2	Prostaglandin E2
PHD	Prolyl hydroxylase
PKB	Protein kinase B
PKM2	Pyruvate kinase M2
PMN-MDSC	Polymorphonuclear myeloid-derived suppressor cell
PPAR-γ	Peroxisome proliferator-activated receptor-γ
PPP	Pentose phosphate pathway
PI3K	Phosphoinositide 3-kinase
SCENITH	Single-cell energetic metabolism by profiling translation
SCF	c-Kit-derived ligand
SDH	Succinate dehydrogenase
Sema3A	Semaphorin 3A
SLC27	Solute carrier family 27
SRA	Scavenger A receptor
SREBP	Sterol regulatory element-binding protein
STAT3	Signal transducer and activator of transcription 6
STAT6	Signal transducer and activator of transcription 6
TAM	Tumor-associated macrophage
TAN	Tumor-associated neutrophil
TBK1	TANK-binding kinase 1
TGFβ	Transforming growth factor β
TIGIT	T cell immunoreceptor with Ig and ITIM domains
TLR	Toll-like receptor
TME	Tumor microenvironment
TNBC	Triple-negative breast cancer
VEGF	Vascular endothelial growth factor
VSIG4	V-set immunoglobulin-domain-containing 4

## References

[1] De Berardinis R.J., Chandel N.S. (2016). Fundamentals of cancer metabolism. Sci. Adv..

[2] Lee N., Kim D. (2016). Cancer metabolism: Fueling more than just growth. Mol. Cells.

[3] DeBerardinis R.J., Lum J.J., Hatzivassiliou G., Thompson C.B. (2008). The Biology of Cancer: Metabolic Reprogramming Fuels Cell Growth and Proliferation. Cell Metab..

[4] Domblides C., Lartigue L., Faustin B. (2019). Control of the Antitumor Immune Response by Cancer Metabolism. Cells.

[5] Biswas S.K. (2015). Metabolic Reprogramming of Immune Cells in Cancer Progression. Immunity.

[6] Kim J. (2018). Regulation of immune cell functions by metabolic reprogramming. J. Immunol. Res..

[7] Wolchok J.D., Chiarion-Sileni V., Gonzalez R., Rutkowski P., Grob J.J., Cowey C.L., Lao C.D., Wagstaff J., Schadendorf D., Ferrucci P.F. (2017). Overall Survival with Combined Nivolumab and Ipilimumab in Advanced Melanoma. N. Engl. J. Med..

[8] Gettinger S., Horn L., Jackman D., Spigel D., Antonia S., Hellmann M., Powderly J., Heist R., Sequist L.V., Smith D.C. (2018). Five-year follow-up of nivolumab in previously treated advanced non–small-cell lung cancer: Results from the CA209-003 study. J. Clin. Oncol..

[9] Le Bourgeois T., Strauss L., Aksoylar H.I., Daneshmandi S., Seth P., Patsoukis N., Boussiotis V.A. (2018). Targeting T cell metabolism for improvement of cancer immunotherapy. Front. Oncol..

[10] Kishton R.J., Sukumar M., Restifo N.P. (2017). Metabolic Regulation of T Cell Longevity and Function in Tumor Immunotherapy. Cell Metab..

[11] Chang C.H., Pearce E.L. (2016). Emerging concepts of T cell metabolism as a target of immunotherapy. Nat. Immunol..

[12] Patel C.H., Powell J.D. (2017). Targeting T cell metabolism to regulate T cell activation, differentiation and function in disease. Curr. Opin. Immunol..

[13] Broz M.L., Binnewies M., Boldajipour B., Nelson A.E., Pollack J.L., Erle D.J., Barczak A., Rosenblum M.D., Daud A., Barber D.L. (2014). Dissecting the Tumor Myeloid Compartment Reveals Rare Activating Antigen-Presenting Cells Critical for T Cell Immunity. Cancer Cell.

[14] Merad M., Sathe P., Helft J., Miller J., Mortha A. (2013). The dendritic cell lineage: Ontogeny and function of dendritic cells and their subsets in the steady state and the inflamed setting. Annu. Rev. Immunol..

[15] Salmon H., Idoyaga J., Rahman A., Leboeuf M., Remark R., Jordan S., Casanova-Acebes M., Khudoynazarova M., Agudo J., Tung N. (2016). Expansion and Activation of CD103+ Dendritic Cell Progenitors at the Tumor Site Enhances Tumor Responses to Therapeutic PD-L1 and BRAF Inhibition. Immunity.

[16] Sánchez-Paulete A.R., Cueto F.J., Martínez-López M., Labiano S., Morales-Kastresana A., Rodríguez-Ruiz M.E., Jure-Kunkel M., Azpilikueta A., Aznar M.A., Quetglas J.I. (2016). Cancer immunotherapy with immunomodulatory anti-CD137 and anti–PD-1 monoclonal antibodies requires BATF3-dependent dendritic cells. Cancer Discov..

[17] Wculek S.K., Amores-Iniesta J., Conde-Garrosa R., Khouili S.C., Melero I., Sancho D. (2019). Effective cancer immunotherapy by natural mouse conventional type-1 dendritic cells bearing dead tumor antigen. J. Immunother. Cancer.

[18] Binnewies M., Mujal A.M., Pollack J.L., Combes A.J., Hardison E.A., Barry K.C., Tsui J., Ruhland M.K., Kersten K., Abushawish M.A. (2019). Unleashing Type-2 Dendritic Cells to Drive Protective Antitumor CD4^+^ T Cell Immunity. Cell.

[19] Demoulin S., Herfs M., Delvenne P., Hubert P. (2013). Tumor microenvironment converts plasmacytoid dendritic cells into immunosuppressive/tolerogenic cells: Insight into the molecular mechanisms. J. Leukoc. Biol..

[20] Butterfield L.H. (2016). Lessons learned from cancer vaccine trials and target antigen choice. Cancer Immunol. Immunother..

[21] Krawczyk C.M., Holowka T., Sun J., Blagih J., Amiel E., DeBerardinis R.J., Cross J.R., Jung E., Thompson C.B., Jones R.G. (2010). Toll-like receptor-induced changes in glycolytic metabolism regulate dendritic cell activation. Blood.

[22] Everts B., Amiel E., Huang S.C.C., Smith A.M., Chang C.H., Lam W.Y., Redmann V., Freitas T.C., Blagih J., Van Der Windt G.J.W. (2014). TLR-driven early glycolytic reprogramming via the kinases TBK1-IKKε supports the anabolic demands of dendritic cell activation. Nat. Immunol..

[23] Everts B., Amiel E., Van Der Windt G.J.W., Freitas T.C., Chott R., Yarasheski K.E., Pearce E.L., Pearce E.J. (2012). Commitment to glycolysis sustains survival of NO-producing inflammatory dendritic cells. Blood.

[24] Thwe P.M., Pelgrom L., Cooper R., Beauchamp S., Reisz J.A., D’Alessandro A., Everts B., Amiel E. (2017). Cell-Intrinsic Glycogen Metabolism Supports Early Glycolytic Reprogramming Required for Dendritic Cell Immune Responses. Cell Metab..

[25] Jantsch J., Chakravortty D., Turza N., Prechtel A.T., Buchholz B., Gerlach R.G., Volke M., Gläsner J., Warnecke C., Wiesener M.S. (2008). Hypoxia and Hypoxia-Inducible Factor-1α Modulate Lipopolysaccharide-Induced Dendritic Cell Activation and Function. J. Immunol..

[26] Guak H., Al Habyan S., Ma E.H., Aldossary H., Al-Masri M., Won S.Y., Ying T., Fixman E.D., Jones R.G., McCaffrey L.M. (2018). Glycolytic metabolism is essential for CCR7 oligomerization and dendritic cell migration. Nat. Commun..

[27] Rubic T., Lametschwandtner G., Jost S., Hinteregger S., Kund J., Carballido-Perrig N., Schwärzler C., Junt T., Voshol H., Meingassner J.G. (2008). Triggering the succinate receptor GPR91 on dendritic cells enhances immunity. Nat. Immunol..

[28] Wu D., Sanin D.E., Everts B., Chen Q., Qiu J., Buck M.D., Patterson A., Smith A.M., Chang C.H., Liu Z. (2016). Type 1 Interferons Induce Changes in Core Metabolism that Are Critical for Immune Function. Immunity.

[29] Lawless S.J., Kedia-Mehta N., Walls J.F., McGarrigle R., Convery O., Sinclair L.V., Navarro M.N., Murray J., Finlay D.K. (2017). Glucose represses dendritic cell-induced T cell responses. Nat. Commun..

[30] Weichhart T., Costantino G., Poglitsch M., Rosner M., Zeyda M., Stuhlmeier K.M., Kolbe T., Stulnig T.M., Hörl W.H., Hengstschläger M. (2008). The TSC-mTOR Signaling Pathway Regulates the Innate Inflammatory Response. Immunity.

[31] Amiel E., Everts B., Freitas T.C., King I.L., Curtis J.D., Pearce E.L., Pearce E.J. (2012). Inhibition of Mechanistic Target of Rapamycin Promotes Dendritic Cell Activation and Enhances Therapeutic Autologous Vaccination in Mice. J. Immunol..

[32] Currie E., Schulze A., Zechner R., Walther T.C., Farese R.V. (2013). Cellular fatty acid metabolism and cancer. Cell Metab..

[33] Böttcher J.P., Bonavita E., Chakravarty P., Blees H., Cabeza-Cabrerizo M., Sammicheli S., Rogers N.C., Sahai E., Zelenay S., Reis e Sousa C. (2018). NK Cells Stimulate Recruitment of cDC1 into the Tumor Microenvironment Promoting Cancer Immune Control. Cell.

[34] Herber D.L., Cao W., Nefedova Y., Novitskiy S.V., Nagaraj S., Tyurin V.A., Corzo A., Cho H.I., Celis E., Lennox B. (2010). Lipid accumulation and dendritic cell dysfunction in cancer. Nat. Med..

[35] Cubillos-Ruiz J.R., Silberman P.C., Rutkowski M.R., Chopra S., Perales-Puchalt A., Song M., Zhang S., Bettigole S.E., Gupta D., Holcomb K. (2015). ER Stress Sensor XBP1 Controls Anti-tumor Immunity by Disrupting Dendritic Cell Homeostasis. Cell.

[36] Christofides A., Konstantinidou E., Jani C., Boussiotis V.A. (2021). The role of peroxisome proliferator-activated receptors (PPAR) in immune responses. Metabolism.

[37] Zhao F., Xiao C., Evans K.S., Theivanthiran T., DeVito N., Holtzhausen A., Liu J., Liu X., Boczkowski D., Nair S. (2018). Paracrine Wnt5a-β-Catenin Signaling Triggers a Metabolic Program that Drives Dendritic Cell Tolerization. Immunity.

[38] Nasi A., Fekete T., Krishnamurthy A., Snowden S., Rajnavölgyi E., Catrina A.I., Wheelock C.E., Vivar N., Rethi B. (2013). Dendritic Cell Reprogramming by Endogenously Produced Lactic Acid. J. Immunol..

[39] Li L., Huang L., Ye H., Song S.P., Bajwa A., Lee S.J., Moser E.K., Jaworska K., Kinsey G.R., Day Y.J. (2012). Dendritic cells tolerized with adenosine A2AR agonist attenuate acute kidney injury. J. Clin. Investig..

[40] Leone R.D., Emens L.A. (2018). Targeting adenosine for cancer immunotherapy. J. Immunother. Cancer.

[41] Murray P.J., Allen J.E., Biswas S.K., Fisher E.A., Gilroy D.W., Goerdt S., Gordon S., Hamilton J.A., Ivashkiv L.B., Lawrence T. (2014). Macrophage Activation and Polarization: Nomenclature and Experimental Guidelines. Immunity.

[42] Kaneda M.M., Cappello P., Nguyen A.V., Ralainirina N., Hardamon C.R., Foubert P., Schmid M.C., Sun P., Mose E., Bouvet M. (2016). Macrophage PI3Kγ drives pancreatic ductal adenocarcinoma progression. Cancer Discov..

[43] Najafi M., Hashemi Goradel N., Farhood B., Salehi E., Nashtaei M.S., Khanlarkhani N., Khezri Z., Majidpoor J., Abouzaripour M., Habibi M. (2019). Macrophage polarity in cancer: A review. J. Cell. Biochem..

[44] Ye H., Zhou Q., Zheng S., Li G., Lin Q., Wei L., Fu Z., Zhang B., Liu Y., Li Z. (2018). Tumor-associated macrophages promote progression and the Warburg effect via CCL18/NF-kB/VCAM-1 pathway in pancreatic ductal adenocarcinoma. Cell Death Dis..

[45] Tan B., Shi X., Zhang J., Qin J., Zhang N., Ren H., Qian M., Siwko S., Carmon K., Liu Q. (2018). Inhibition of RSPO-LGR4 facilitates checkpoint blockade therapy by switching macrophage polarization. Cancer Res..

[46] Freemerman A.J., Johnson A.R., Sacks G.N., Milner J.J., Kirk E.L., Troester M.A., Macintyre A.N., Goraksha-Hicks P., Rathmell J.C., Makowski L. (2014). Metabolic reprogramming of macrophages: Glucose transporter 1 (GLUT1)-mediated glucose metabolism drives a proinflammatory phenotype. J. Biol. Chem..

[47] Naik A., Monjazeb A.M., Decock J. (2019). The obesity paradox in cancer, tumor immunology, and immunotherapy: Potential therapeutic implications in triple negative breast cancer. Front. Immunol..

[48] Cortellini A., Bersanelli M., Buti S., Cannita K., Santini D., Perrone F., Giusti R., Tiseo M., Michiara M., Di Marino P. (2019). A multicenter study of body mass index in cancer patients treated with anti-PD-1/PD-L1 immune checkpoint inhibitors: When overweight becomes favorable. J. Immunother. Cancer.

[49] Newsholme P., Curi R., Gordon S., Newsholme E.A. (1986). Metabolism of glucose, glutamine, long-chain fatty acids and ketone bodies by murine macrophages. Biochem. J..

[50] Gamelli R.L., Liu H., He L.K., Hofmann C.A. (1996). Augmentations of glucose uptake and glucose transporter-1 in macrophages following thermal injury and sepsis in mice. J. Leukoc. Biol..

[51] Moon J.S., Hisata S., Park M.A., DeNicola G.M., Ryter S.W., Nakahira K., Choi A.M.K. (2015). MTORC1-Induced HK1-Dependent Glycolysis Regulates NLRP3 Inflammasome Activation. Cell Rep..

[52] Deng W., Yang J., Lin X., Shin J., Gao J., Zhong X.-P. (2017). Essential Role of mTORC1 in Self-Renewal of Murine Alveolar Macrophages. J. Immunol..

[53] Haschemi A., Kosma P., Gille L., Evans C.R., Burant C.F., Starkl P., Knapp B., Haas R., Schmid J.A., Jandl C. (2012). The sedoheptulose kinase CARKL directs macrophage polarization through control of glucose metabolism. Cell Metab..

[54] Vats D., Mukundan L., Odegaard J.I., Zhang L., Smith K.L., Morel C.R., Greaves D.R., Murray P.J., Chawla A. (2006). Oxidative metabolism and PGC-1β attenuate macrophage-mediated inflammation. Cell Metab..

[55] Sag D., Carling D., Stout R.D., Suttles J. (2008). Adenosine 5′-Monophosphate-Activated Protein Kinase Promotes Macrophage Polarization to an Anti-Inflammatory Functional Phenotype. J. Immunol..

[56] Tannahill G.M., Curtis A.M., Adamik J., Palsson-Mcdermott E.M., McGettrick A.F., Goel G., Frezza C., Bernard N.J., Kelly B., Foley N.H. (2013). Succinate is an inflammatory signal that induces IL-1β through HIF-1α. Nature.

[57] Mills E.L., Kelly B., Logan A., Costa A.S.H., Varma M., Bryant C.E., Tourlomousis P., Däbritz J.H.M., Gottlieb E., Latorre I. (2016). Succinate Dehydrogenase Supports Metabolic Repurposing of Mitochondria to Drive Inflammatory Macrophages. Cell.

[58] Cordes T., Wallace M., Michelucci A., Divakaruni A.S., Sapcariu S.C., Sousa C., Koseki H., Cabrales P., Murphy A.N., Hiller K. (2016). Immunoresponsive gene 1 and itaconate inhibit succinate dehydrogenase to modulate intracellular succinate levels. J. Biol. Chem..

[59] Lampropoulou V., Sergushichev A., Bambouskova M., Nair S., Vincent E.E., Loginicheva E., Cervantes-Barragan L., Ma X., Huang S.C.C., Griss T. (2016). Itaconate Links Inhibition of Succinate Dehydrogenase with Macrophage Metabolic Remodeling and Regulation of Inflammation. Cell Metab..

[60] Strelko C.L., Lu W., Dufort F.J., Seyfried T.N., Chiles T.C., Rabinowitz J.D., Roberts M.F. (2011). Itaconic acid is a mammalian metabolite induced during macrophage activation. J. Am. Chem. Soc..

[61] Sugimoto M., Sakagami H., Yokote Y., Onuma H., Kaneko M., Mori M., Sakaguchi Y., Soga T., Tomita M. (2012). Non-targeted metabolite profiling in activated macrophage secretion. Metabolomics.

[62] Weiss J.M., Davies L.C., Karwan M., Ileva L., Ozaki M.K., Cheng R.Y.S., Ridnour L.A., Annunziata C.M., Wink D.A., McVicar D.W. (2018). Itaconic acid mediates crosstalk between macrophage metabolism and peritoneal tumors. J. Clin. Investig..

[63] Liu P.S., Wang H., Li X., Chao T., Teav T., Christen S., DI Conza G., Cheng W.C., Chou C.H., Vavakova M. (2017). α-ketoglutarate orchestrates macrophage activation through metabolic and epigenetic reprogramming. Nat. Immunol..

[64] Casazza A., Laoui D., Wenes M., Rizzolio S., Bassani N., Mambretti M., Deschoemaeker S., VanGinderachter J.A., Tamagnone L., Mazzone M. (2013). Impeding Macrophage Entry into Hypoxic Tumor Areas by Sema3A/Nrp1 Signaling Blockade Inhibits Angiogenesis and Restores Antitumor Immunity. Cancer Cell.

[65] Tripathi C., Tewari B.N., Kanchan R.K., Baghel K.S., Nautiyal N., Shrivastava R., Kaur H., Bramha Bhatt M.L., Bhadauria S. (2014). Macrophages are recruited to hypoxic tumor areas and acquire a Pro-Angiogenic M2-Polarized phenotype via hypoxic cancer cell derived cytokines Oncostatin M and Eotaxin. Oncotarget.

[66] Colegio O.R., Chu N.Q., Szabo A.L., Chu T., Rhebergen A.M., Jairam V., Cyrus N., Brokowski C.E., Eisenbarth S.C., Phillips G.M. (2014). Functional polarization of tumour-associated macrophages by tumour-derived lactic acid. Nature.

[67] Palsson-McDermott E.M., Dyck L., Zaslona Z., Menon D., McGettrick A.F., Mills K.H.G., O’Neill L.A. (2017). Pyruvate kinase M2 is required for the expression of the immune checkpoint PD-L1 in immune cells and tumors. Front. Immunol..

[68] Li J., Diao B., Guo S., Huang X., Yang C., Feng Z., Yan W., Ning Q., Zheng L., Chen Y. (2017). VSIG4 inhibits proinflammatory macrophage activation by reprogramming mitochondrial pyruvate metabolism. Nat. Commun..

[69] Rath M., Müller I., Kropf P., Closs E.I., Munder M. (2014). Metabolism via arginase or nitric oxide synthase: Two competing arginine pathways in macrophages. Front. Immunol..

[70] El-Gayar S., Thüring-Nahler H., Pfeilschifter J., Röllinghoff M., Bogdan C. (2003). Translational Control of Inducible Nitric Oxide Synthase by IL-13 and Arginine Availability in Inflammatory Macrophages. J. Immunol..

[71] Jha A.K., Huang S.C.C., Sergushichev A., Lampropoulou V., Ivanova Y., Loginicheva E., Chmielewski K., Stewart K.M., Ashall J., Everts B. (2015). Network integration of parallel metabolic and transcriptional data reveals metabolic modules that regulate macrophage polarization. Immunity.

[72] Palmieri E.M., Menga A., Martín-Pérez R., Quinto A., Riera-Domingo C., De Tullio G., Hooper D.C., Lamers W.H., Ghesquière B., McVicar D.W. (2017). Pharmacologic or Genetic Targeting of Glutamine Synthetase Skews Macrophages toward an M1-like Phenotype and Inhibits Tumor Metastasis. Cell Rep..

[73] Csóka B., Selmeczy Z., Koscsó B., Németh Z.H., Pacher P., Murray P.J., Kepka-Lenhart D., Morris S.M., Gause W.C., Leibovich S.J. (2012). Adenosine promotes alternative macrophage activation via A2A and A2B receptors. FASEB J..

[74] Ferrante C.J., Pinhal-Enfield G., Elson G., Cronstein B.N., Hasko G., Outram S., Leibovich S.J. (2013). The adenosine-dependent angiogenic switch of macrophages to an M2-like phenotype is independent of interleukin-4 receptor alpha (IL-4Rα) signaling. Inflammation.

[75] Bronte V., Brandau S., Chen S.H., Colombo M.P., Frey A.B., Greten T.F., Mandruzzato S., Murray P.J., Ochoa A., Ostrand-Rosenberg S. (2016). Recommendations for myeloid-derived suppressor cell nomenclature and characterization standards. Nat. Commun..

[76] Veglia F., Perego M., Gabrilovich D. (2018). Myeloid-derived suppressor cells coming of age. Nat. Immunol..

[77] Wang Y., Ding Y., Guo N., Wang S. (2019). MDSCs: Key criminals of tumor pre-metastatic niche formation. Front. Immunol..

[78] Umansky V., Blattner C., Gebhardt C., Utikal J. (2016). The role of myeloid-derived suppressor cells (MDSC) in cancer progression. Vaccines.

[79] Hossain F., Al-Khami A.A., Wyczechowska D., Hernandez C., Zheng L., Reiss K., Del Valle L., Trillo-Tinoco J., Maj T., Zou W. (2015). Inhibition of Fatty Acid Oxidation Modulates Immunosuppressive Functions of Myeloid-Derived Suppressor Cells and Enhances Cancer Therapies. Cancer Immunol. Res..

[80] Coffelt S.B., Wellenstein M.D., De Visser K.E. (2016). Neutrophils in cancer: Neutral no more. Nat. Rev. Cancer.

[81] Templeton A.J., McNamara M.G., Šeruga B., Vera-Badillo F.E., Aneja P., Ocaña A., Leibowitz-Amit R., Sonpavde G., Knox J.J., Tran B. (2014). Prognostic role of neutrophil-to-lymphocyte ratio in solid tumors: A systematic review and meta-analysis. J. Natl. Cancer Inst..

[82] Michaeli J., Shaul M.E., Mishalian I., Hovav A.H., Levy L., Zolotriov L., Granot Z., Fridlender Z.G. (2017). Tumor-associated neutrophils induce apoptosis of non-activated CD8 T-cells in a TNFα and NO-dependent mechanism, promoting a tumor-supportive environment. Oncoimmunology.

[83] Liang W., Ferrara N. (2016). The complex role of Neutrophils in tumor angiogenesis and metastasis. Cancer Immunol. Res..

[84] Garley M., Jabłońska E., Dąbrowska D. (2016). NETs in cancer. Tumor Biol..

[85] Oklu R., Sheth R.A., Wong K.H.K., Jahromi A.H., Albadawi H. (2017). Neutrophil extracellular traps are increased in cancer patients but does not associate with venous thrombosis. Cardiovasc. Diagn. Ther..

[86] Vols S., Sionov R.V., Granot Z. (2017). Always Look On the Bright Side: Anti-Tumor Functions of Neutrophils. Curr. Pharm. Des..

[87] Chacko B.K., Kramer P.A., Ravi S., Johnson M.S., Hardy R.W., Ballinger S.W., Darley-Usmar V.M. (2013). Methods for defining distinct bioenergetic profiles in platelets, lymphocytes, monocytes, and neutrophils, and the oxidative burst from human blood. Lab. Investig..

[88] Sadiku P., Willson J.A., Dickinson R.S., Murphy F., Harris A.J., Lewis A., Sammut D., Mirchandani A.S., Ryan E., Watts E.R. (2017). Prolyl hydroxylase 2 inactivation enhances glycogen storage and promotes excessive neutrophilic responses. J. Clin. Investig..

[89] Rodríguez-Espinosa O., Rojas-Espinosa O., Moreno-Altamirano M.M.B., López-Villegas E.O., Sánchez-García F.J. (2015). Metabolic requirements for neutrophil extracellular traps formation. Immunology.

[90] Azevedo E.P., Rochael N.C., Guimarães-Costa A.B., De Souza-Vieira T.S., Ganilho J., Saraiva E.M., Palhano F.L., Foguel D. (2015). A metabolic shift toward pentose phosphate pathway is necessary for amyloid fibril- and phorbol 12-myristate 13-Acetate-induced neutrophil extracellular trap (NET) formation. J. Biol. Chem..

[91] Hsu B.E., Tabariès S., Johnson R.M., Andrzejewski S., Senecal J., Lehuédé C., Annis M.G., Ma E.H., Völs S., Ramsay L.A. (2019). Immature Low-Density Neutrophils Exhibit Metabolic Flexibility that Facilitates Breast Cancer Liver Metastasis. Cell Rep..

[92] Jian S.L., Chen W.W., Su Y.C., Su Y.W., Chuang T.H., Hsu S.C., Huang L.R. (2017). Glycolysis regulates the expansion of myeloid-derived suppressor cells in tumor-bearing hosts through prevention of ROS-mediated apoptosis. Cell Death Dis..

[93] Li W., Tanikawa T., Kryczek I., Xia H., Li G., Wu K., Wei S., Zhao L., Vatan L., Wen B. (2018). Aerobic Glycolysis Controls Myeloid-Derived Suppressor Cells and Tumor Immunity via a Specific CEBPB Isoform in Triple-Negative Breast Cancer. Cell Metab..

[94] Husain Z., Huang Y., Seth P., Sukhatme V.P. (2013). Tumor-Derived Lactate Modifies Antitumor Immune Response: Effect on Myeloid-Derived Suppressor Cells and NK Cells. J. Immunol..

[95] Ye C., Geng Z., Dominguez D., Chen S., Fan J., Qin L., Long A., Zhang Y., Kuzel T.M., Zhang B. (2016). Targeting Ornithine Decarboxylase by α-Difluoromethylornithine Inhibits Tumor Growth by Impairing Myeloid-Derived Suppressor Cells. J. Immunol..

[96] Reczkowski R.S., Ash D.E. (1994). Rat liver arginase: Kinetic mechanism, alternate substrates, and inhibitors. Arch. Biochem. Biophys..

[97] Corzo C.A., Condamine T., Lu L., Cotter M.J., Youn J.I., Cheng P., Cho H.I., Celis E., Quiceno D.G., Padhya T. (2010). HIF-1α regulates function and differentiation of myeloid-derived suppressor cells in the tumor microenvironment. J. Exp. Med..

[98] Noman M.Z., Desantis G., Janji B., Hasmim M., Karray S., Dessen P., Bronte V., Chouaib S. (2014). PD-L1 is a novel direct target of HIF-1α, and its blockade under hypoxia enhanced: MDSC-mediated T cell activation. J. Exp. Med..

[99] Patel S., Fu S., Mastio J., Dominguez G.A., Purohit A., Kossenkov A., Lin C., Alicea-Torres K., Sehgal M., Nefedova Y. (2018). Unique pattern of neutrophil migration and function during tumor progression. Nat. Immunol..

[100] Rice C.M., Davies L.C., Subleski J.J., Maio N., Gonzalez-Cotto M., Andrews C., Patel N.L., Palmieri E.M., Weiss J.M., Lee J.M. (2018). Tumour-elicited neutrophils engage mitochondrial metabolism to circumvent nutrient limitations and maintain immune suppression. Nat. Commun..

[101] Veglia F., Tyurin V.A., Blasi M., De Leo A., Kossenkov A.V., Donthireddy L., To T.K.J., Schug Z., Basu S., Wang F. (2019). Fatty acid transport protein 2 reprograms neutrophils in cancer. Nature.

[102] Rotondo R., Barisione G., Mastracci L., Grossi F., Orengo A.M., Costa R., Truini M., Fabbi M., Ferrini S., Barbieri O. (2009). IL-8 induces exocytosis of arginase 1 by neutrophil polymorphonuclears in nonsmall cell lung cancer. Int. J. Cancer.

[103] Borek B., Gajda T., Golebiowski A., Blaszczyk R. (2020). Boronic acid-based arginase inhibitors in cancer immunotherapy. Bioorgan. Med. Chem..

[104] Zelenay S., Van Der Veen A.G., Böttcher J.P., Snelgrove K.J., Rogers N., Acton S.E., Chakravarty P., Girotti M.R., Marais R., Quezada S.A. (2015). Cyclooxygenase-Dependent Tumor Growth through Evasion of Immunity. Cell.

[105] Steggerda S.M., Bennett M.K., Chen J., Emberley E., Huang T., Janes J.R., Li W., MacKinnon A.L., Makkouk A., Marguier G. (2017). Inhibition of arginase by CB-1158 blocks myeloid cell-mediated immune suppression in the tumor microenvironment. J. Immunother. Cancer.

[106] Naing A., Bauer T., Papadopoulos K.P., Rahma O., Tsai F., Garralda E., Naidoo J., Pai S., Gibson M.K., Rybkin I. (2019). Phase I study of the arginase inhibitor INCB001158 (1158) alone and in combination with pembrolizumab (PEM) in patients (Pts) with advanced/metastatic (adv/met) solid tumours. Ann. Oncol..

[107] Shimasaki N., Jain A., Campana D. (2020). NK cells for cancer immunotherapy. Nat. Rev. Drug Discov..

[108] Albertsson P.A., Basse P.H., Hokland M., Goldfarb R.H., Nagelkerke J.F., Nannmark U., Kuppen P.J.K. (2003). NK cells and the tumour microenvironment: Implications for NK-cell function and anti-tumour activity. Trends Immunol..

[109] Alvarez I.B., Pasquinelli V., Jurado J.O., Abbate E., Musella R.M., De La Barrera S.S., García V.E. (2010). Role played by the programmed death-1-programmed death ligand pathway during innate immunity against Mycobacterium tuberculosis. J. Infect. Dis..

[110] Norris S., Coleman A., Kuri-Cervantes L., Bower M., Nelson M., Goodier M.R. (2012). PD-1 expression on natural killer cells and CD8+ T cells during chronic HIV-1 infection. Viral Immunol..

[111] Stojanovic A., Fiegler N., Brunner-Weinzierl M., Cerwenka A. (2014). CTLA-4 Is Expressed by Activated Mouse NK Cells and Inhibits NK Cell IFN-γ Production in Response to Mature Dendritic Cells. J. Immunol..

[112] Zhang Q., Bi J., Zheng X., Chen Y., Wang H., Wu W., Wang Z., Wu Q., Peng H., Wei H. (2018). Blockade of the checkpoint receptor TIGIT prevents NK cell exhaustion and elicits potent anti-tumor immunity. Nat. Immunol..

[113] Vari F., Arpon D., Keane C., Hertzberg M.S., Talaulikar D., Jain S., Cui Q., Han E., Tobin J., Bird R. (2018). Immune evasion via PD-1/PD-L1 on NK cells and monocyte/macrophages is more prominent in Hodgkin lymphoma than DLBCL. Blood.

[114] Hsu J., Hodgins J.J., Marathe M., Nicolai C.J., Bourgeois-Daigneault M.C., Trevino T.N., Azimi C.S., Scheer A.K., Randolph H.E., Thompson T.W. (2018). Contribution of NK cells to immunotherapy mediated by PD-1/PD-L1 blockade. J. Clin. Investig..

[115] André P., Denis C., Soulas C., Bourbon-Caillet C., Lopez J., Arnoux T., Bléry M., Bonnafous C., Gauthier L., Morel A. (2018). Anti-NKG2A mAb Is a Checkpoint Inhibitor that Promotes Anti-tumor Immunity by Unleashing Both T and NK Cells. Cell.

[116] Klingemann H. (2014). Are natural killer cells superior CAR drivers?. Oncoimmunology.

[117] Marçais A., Cherfils-Vicini J., Viant C., Degouve S., Viel S., Fenis A., Rabilloud J., Mayol K., Tavares A., Bienvenu J. (2014). The metabolic checkpoint kinase mTOR is essential for IL-15 signaling during the development and activation of NK cells. Nat. Immunol..

[118] Keppel M.P., Saucier N., Mah A.Y., Vogel T.P., Cooper M.A. (2015). Activation-Specific Metabolic Requirements for NK Cell IFN-γ Production. J. Immunol..

[119] Donnelly R.P., Loftus R.M., Keating S.E., Liou K.T., Biron C.A., Gardiner C.M., Finlay D.K. (2014). mTORC1-Dependent Metabolic Reprogramming Is a Prerequisite for NK Cell Effector Function. J. Immunol..

[120] Viel S., Marçais A., Guimaraes F.S.F., Loftus R., Rabilloud J., Grau M., Degouve S., Djebali S., Sanlaville A., Charrier E. (2016). TGF-β inhibits the activation and functions of NK cells by repressing the mTOR pathway. Sci. Signal..

[121] Michelet X., Dyck L., Hogan A., Loftus R.M., Duquette D., Wei K., Beyaz S., Tavakkoli A., Foley C., Donnelly R. (2018). Metabolic reprogramming of natural killer cells in obesity limits antitumor responses. Nat. Immunol..

[122] Loftus R.M., Assmann N., Kedia-Mehta N., O’Brien K.L., Garcia A., Gillespie C., Hukelmann J.L., Oefner P.J., Lamond A.I., Gardiner C.M. (2018). Amino acid-dependent cMyc expression is essential for NK cell metabolic and functional responses in mice. Nat. Commun..

[123] Assmann N., O’Brien K.L., Donnelly R.P., Dyck L., Zaiatz-Bittencourt V., Loftus R.M., Heinrich P., Oefner P.J., Lynch L., Gardiner C.M. (2017). Srebp-controlled glucose metabolism is essential for NK cell functional responses. Nat. Immunol..

[124] Li D., Long W., Huang R., Chen Y., Xia M. (2018). 27-Hydroxycholesterol Inhibits Sterol Regulatory Element-Binding Protein 1 Activation and Hepatic Lipid Accumulation in Mice. Obesity.

[125] Wu Q., Ishikawa T., Sirianni R., Tang H., McDonald J.G., Yuhanna I.S., Thompson B., Girard L., Mineo C., Brekken R.A. (2013). 27-Hydroxycholesterol promotes cell-autonomous, ER-positive breast cancer growth. Cell Rep..

[126] Rossin D., Dias I.H.K., Solej M., Milic I., Pitt A.R., Iaia N., Scoppapietra L., Devitt A., Nano M., Degiuli M. (2019). Increased production of 27-hydroxycholesterol in human colorectal cancer advanced stage: Possible contribution to cancer cell survival and infiltration. Free Radic. Biol. Med..

[127] Cong J., Wang X., Zheng X., Wang D., Fu B., Sun R., Tian Z., Wei H. (2018). Dysfunction of Natural Killer Cells by FBP1-Induced Inhibition of Glycolysis during Lung Cancer Progression. Cell Metab..

[128] Brand A., Singer K., Koehl G.E., Kolitzus M., Schoenhammer G., Thiel A., Matos C., Bruss C., Klobuch S., Peter K. (2016). LDHA-Associated Lactic Acid Production Blunts Tumor Immunosurveillance by T and NK Cells. Cell Metab..

[129] Harmon C., Robinson M.W., Hand F., Almuaili D., Mentor K., Houlihan D.D., Hoti E., Lynch L., Geoghegan J., O’Farrelly C. (2019). Lactate-mediated acidification of tumor microenvironment induces apoptosis of liver-resident NK cells in colorectal liver metastasis. Cancer Immunol. Res..

[130] Pilon-Thomas S., Kodumudi K.N., El-Kenawi A.E., Russell S., Weber A.M., Luddy K., Damaghi M., Wojtkowiak J.W., Mulé J.J., Ibrahim-Hashim A. (2016). Neutralization of tumor acidity improves antitumor responses to immunotherapy. Cancer Res..

[131] Balsamo M., Manzini C., Pietra G., Raggi F., Blengio F., Mingari M.C., Varesio L., Moretta L., Bosco M.C., Vitale M. (2013). Hypoxia downregulates the expression of activating receptors involved in NK-cell-mediated target cell killing without affecting ADCC. Eur. J. Immunol..

[132] Krzywinska E., Kantari-Mimoun C., Kerdiles Y., Sobecki M., Isagawa T., Gotthardt D., Castells M., Haubold J., Millien C., Viel T. (2017). Loss of HIF-1α in natural killer cells inhibits tumour growth by stimulating non-productive angiogenesis. Nat. Commun..

[133] Lamas B., Vergnaud-Gauduchon J., Goncalves-Mendes N., Perche O., Rossary A., Vasson M.P., Farges M.C. (2012). Altered functions of natural killer cells in response to L-Arginine availability. Cell. Immunol..

[134] Oberlies J., Watzl C., Giese T., Luckner C., Kropf P., Müller I., Ho A.D., Munder M. (2009). Regulation of NK Cell Function by Human Granulocyte Arginase. J. Immunol..

[135] Young A., Ngiow S.F., Gao Y., Patch A.M., Barkauskas D.S., Messaoudene M., Lin G., Coudert J.D., Stannard K.A., Zitvogel L. (2018). A2AR adenosine signaling suppresses natural killer cell maturation in the tumor microenvironment. Cancer Res..

[136] Mazumdar C., Driggers E.M., Turka L.A. (2020). The Untapped Opportunity and Challenge of Immunometabolism: A New Paradigm for Drug Discovery. Cell Metab..

[137] Voorde J.V., Ackermann T., Pfetzer N., Sumpton D., Mackay G., Kalna G., Nixon C., Blyth K., Gottlieb E., Tardito S. (2019). Improving the metabolic fidelity of cancer models with a physiological cell culture medium. Sci. Adv..

[138] Langhans S.A. (2018). Three-dimensional in vitro cell culture models in drug discovery and drug repositioning. Front. Pharmacol..

[139] Mestas J., Hughes C.C.W. (2004). Of Mice and Not Men: Differences between Mouse and Human Immunology. J. Immunol..

[140] Llufrio E.M., Wang L., Naser F.J., Patti G.J. (2018). Sorting cells alters their redox state and cellular metabolome. Redox Biol..

[141] Dietmair S., Timmins N.E., Gray P.P., Nielsen L.K., Krömer J.O. (2010). Towards quantitative metabolomics of mammalian cells: Development of a metabolite extraction protocol. Anal. Biochem..

[142] Viollet B., Guigas B., Leclerc J., Hébrard S., Lantier L., Mounier R., Andreelli F., Foretz M. (2009). AMP-activated protein kinase in the regulation of hepatic energy metabolism: From physiology to therapeutic perspectives. Acta Physiol..

[143] Dowling R.J.O., Goodwin P.J., Stambolic V. (2011). Understanding the benefit of metformin use in cancer treatment. BMC Med..

[144] Cha J.H., Yang W.H., Xia W., Wei Y., Chan L.C., Lim S.O., Li C.W., Kim T., Chang S.S., Lee H.H. (2018). Metformin Promotes Antitumor Immunity via Endoplasmic-Reticulum-Associated Degradation of PD-L1. Mol. Cell.

[145] Zhang J.-J., Zhang Q.-S., Li Z.-Q., Zhou J.-W., Du J. (2019). Metformin attenuates PD-L1 expression through activating Hippo signaling pathway in colorectal cancer cells. Am. J. Transl. Res..

[146] Scharping N.E., Menk A.V., Whetstone R.D., Zeng X., Delgoffe G.M. (2017). Efficacy of PD-1 blockade is potentiated by metformin-induced reduction of tumor hypoxia. Cancer Immunol. Res..

[147] Ren D., Qin G., Zhao J., Sun Y., Zhang B., Li D., Wang B., Jin X., Wu H. (2020). Metformin activates the STING/IRF3/IFN-β pathway by inhibiting AKT phosphorylation in pancreatic cancer. Am. J. Cancer Res..

[148] Bunt S.K., Mohr A.M., Bailey J.M., Grandgenett P.M., Hollingsworth M.A. (2013). Rosiglitazone and Gemcitabine in combination reduces immune suppression and modulates T cell populations in pancreatic cancer. Cancer Immunol. Immunother..

[149] Ishay-Ronen D., Diepenbruck M., Kalathur R.K.R., Sugiyama N., Tiede S., Ivanek R., Bantug G., Morini M.F., Wang J., Hess C. (2019). Gain Fat—Lose Metastasis: Converting Invasive Breast Cancer Cells into Adipocytes Inhibits Cancer Metastasis. Cancer Cell.

[150] Varghese S., Pramanik S., Williams L.J., Hodges H.R., Hudgens C.W., Fischer G.M., Luo C.K., Knighton B., Tan L., Lorenzi P.L. (2020). The glutaminase inhibitor CB-839 (Telaglenastat) enhances the anti-melanoma activity of T cell mediated immunotherapies. Mol. Cancer Ther..

[151] Long G.V., Dummer R., Hamid O., Gajewski T.F., Caglevic C., Dalle S., Arance A., Carlino M.S., Grob J.J., Kim T.M. (2019). Epacadostat plus pembrolizumab versus placebo plus pembrolizumab in patients with unresectable or metastatic melanoma (ECHO-301/KEYNOTE-252): A phase 3, randomised, double-blind study. Lancet Oncol..

[152] Hartmann F.J., Bendall S.C. (2020). Immune monitoring using mass cytometry and related high-dimensional imaging approaches. Nat. Rev. Rheumatol..

[153] Argüello R.J., Combes A.J., Char R., Gigan J.P., Baaziz A.I., Bousiquot E., Camosseto V., Samad B., Tsui J., Yan P. (2020). SCENITH: A Flow Cytometry-Based Method to Functionally Profile Energy Metabolism with Single-Cell Resolution. Cell Metab..

[154] Gong Y., Ji P., Yang Y.S., Xie S., Yu T.J., Xiao Y., Jin M.L., Ma D., Guo L.W., Pei Y.C. (2021). Metabolic-Pathway-Based Subtyping of Triple-Negative Breast Cancer Reveals Potential Therapeutic Targets. Cell Metab..

[155] Sukumar M., Liu J., Ji Y., Subramanian M., Crompton J.G., Yu Z., Roychoudhuri R., Palmer D.C., Muranski P., Karoly E.D. (2013). Inhibiting glycolytic metabolism enhances CD8+ T cell memory and antitumor function. J. Clin. Investig..

[156] Crompton J.G., Sukumar M., Roychoudhuri R., Clever D., Gros A., Eil R.L., Tran E., Hanada K.I., Yu Z., Palmer D.C. (2015). Akt inhibition enhances expansion of potent tumor-specific lymphocytes with memory cell characteristics. Cancer Res..

